# Effect of essential fatty acids on glucose-induced cytotoxicity to retinal vascular endothelial cells

**DOI:** 10.1186/1476-511X-11-90

**Published:** 2012-07-10

**Authors:** Junhui Shen, Shengrong Shen, Undurti N Das, Guotong Xu

**Affiliations:** 1 Laboratory of Clinical Visual Science, Tongji Eye institute, Tongji University School of Medicine, 1239 Siping Road, Shanghai, 200092, China; 2 Department of Food Science and Nutrition, School of Biosystems Engineering & Food Science, Zhejiang University, Hangzhou, 310058, China; 3 UND Life Sciences, 13800 Fairhill Road, #321, Shaker Heights, OH, 44120, USA; 4 School of Biotechnology, Jawaharlal Nehru Technological University, Kakinada, 533 003, India; 5 Bio-Science Research Centre, Gayatri Vidya Parishad College of Engineering, Visakhapatnam, 530 048, India; 6 Institute of Nutrition Science, Tongji University, Shanghai, 200092, China

**Keywords:** α-linolenic acid, Diabetic retinopathy, Oxidative stress, Membrane fluidity

## Abstract

**Background:**

Diabetic retinopathy is a major complication of dysregulated hyperglycemia. Retinal vascular endothelial cell dysfunction is an early event in the pathogenesis of diabetic retinopathy. Studies showed that hyperglycemia-induced excess proliferation of retinal vascular endothelial cells can be abrogated by docosahexaenoic acid (DHA, 22:6 ω-3) and eicosapentaenoic acid (EPA, 20:5 ω-3). The influence of dietary omega-3 PUFA on brain zinc metabolism has been previously implied. Zn^2+^ is essential for the activity of Δ^6^ desaturase as a co-factor that, in turn, converts essential fatty acids to their respective long chain metabolites. Whether essential fatty acids (EFAs) α-linolenic acid and linoleic acid have similar beneficial effect remains poorly understood.

**Methods:**

RF/6A cells were treated with different concentrations of high glucose, α-linolenic acid and linoleic acid and Zn^2+^. The alterations in mitochondrial succinate dehydrogenase enzyme activity, cell membrane fluidity, reactive oxygen species generation, SOD enzyme and vascular endothelial growth factor (VEGF) secretion were evaluated.

**Results:**

Studies showed that hyperglycemia-induced excess proliferation of retinal vascular endothelial cells can be abrogated by both linoleic acid (LA) and α-linolenic acid (ALA), while the saturated fatty acid, palmitic acid was ineffective. A dose–response study with ALA showed that the activity of the mitochondrial succinate dehydrogenase enzyme was suppressed at all concentrations of glucose tested to a significant degree. High glucose enhanced fluorescence polarization and microviscocity reverted to normal by treatment with Zn^2+^ and ALA. ALA was more potent that Zn^2+^. Increased level of high glucose caused slightly increased ROS generation that correlated with corresponding decrease in SOD activity. ALA suppressed ROS generation to a significant degree in a dose dependent fashion and raised SOD activity significantly. ALA suppressed high-glucose-induced VEGF secretion by RF/6A cells.

**Conclusions:**

These results suggest that EFAs such as ALA and LA may have beneficial action in the prevention of high glucose-induced cellular damage.

## Background

High blood glucose levels in diabetic patients increase the risk of diabetic microangiopathy. One of the earliest abnormalities of diabetic retinopathy is thickening of retinal capillary basement membrane. High glucose levels are capable of altering homeostasis of vascular endothelial cells and lead to changes in gene expression that might initiate diabetic retinopathy [1]. Other important early functional changes in diabetic retinopathy include: increased permeability, which can be attributed to endothelial cell dysfunction and basement membrane leakiness [2]. High glucose significantly enhances the migration of retinal endothelial cells without impacting their proliferation, apoptosis, adhesion, and capillary morphogenesis that seems to occur as a result of increased oxidative stress under high-glucose conditions [3]. High-glucose conditions can produce sustained activation of the downstream prosurvival and promigratory signaling pathways, including Src kinase, phosphatidylinositol 3-kinase/Akt1/endothelial nitric oxide synthase, and ERKs that are essential for enhanced migration of retinal EC [3]. Thus, exposure of retinal endothelial cells to high glucose promotes a promigratory phenotype that contributes to the development of diabetic retinopathy. Hyperglycemia induces an increase in protein kinase C activity in cultured bovine retinal capillary endothelial cells that could enhance neovascularization and cell growth that leads to the development of diabetic vascular complications [4].

Positive effects of fish oil on inflammatory gene expression in the eye have been previously shown, but no direct evidence has been provided for the cytoprotective action of PUFAs on retinal vascular endothelial cells [5]. Previously, we showed that some PUFAs possess cytoprotective actions. For instance, AA, EPA and DHA prevented cytotoxic action of alloxan against pancreatic β cells both *in vitro* and *in vivo*[6-10], which prompted us to evaluate the effect of various PUFAs against glucose-induced cytotoxicity to retinal vascular endothelial cells. The influence of dietary omega-3 PUFAs on brain zinc metabolism has been previously implied [11]. Hence, in the present study we also studied possible influence of Zn^2+^, which is a co-factor that is essential for the activity of Δ^6^ desaturase that converts essential fatty acids (EFAs): linoleic acid (18:2 , n-6, LA) and α-linolenic acid (18:3, n-3, ALA) to their respective long chain metabolites [12-15], on glucose-induced cytotoxicity to retinal vascular endothelial cells in the presence of LA and ALA.

## Results

### Effect of various concentrations of glucose on the proliferation of RF/6A cells in vitro at different time periods

**Figure 1:**
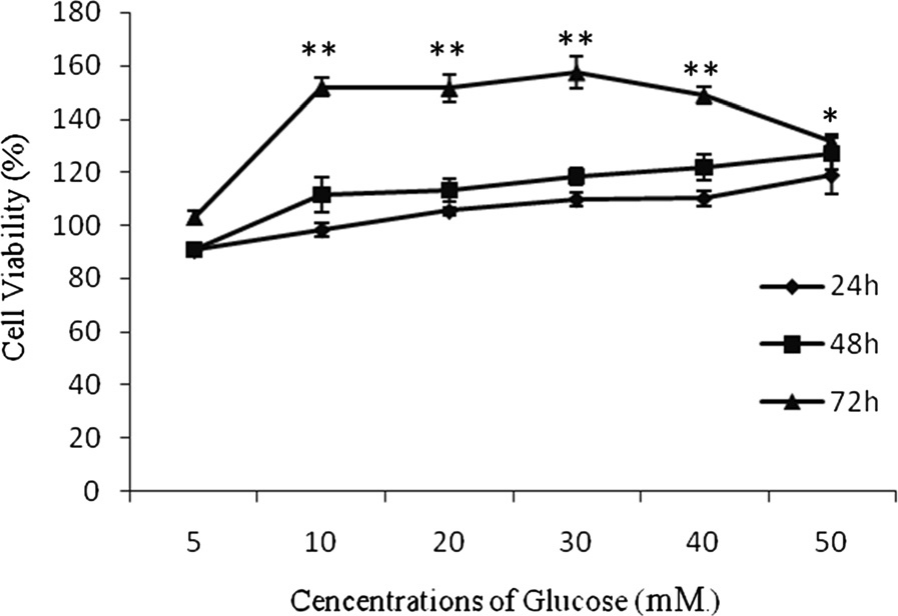
**Effect of various concentrations of glucose on the proliferation of RF/6A cells in vitro at different time periods.** Data are means ± S.D. *P < 0.05 compared to control; **P < 0.05 compared to control.

The effect of various doses of glucose on the proliferation of RF/6A cells were evaluated in a time course study carried out for 24-72hours. The results from MTT assay (Figure 1) showed that glucose promoted cell proliferation at all the concentrations (10 to 50 mM) tested without any effect on their viability and at all time periods tested (24–72 hours). But, the proliferation of cells was maximum (72 > 48 > 24 hours) and statistically significant only at the end of 72 hours of incubation.

### Effect of fatty acids on glucose-induced changes on the proliferation of RF/6A cells in vitro

**Figure 2:**
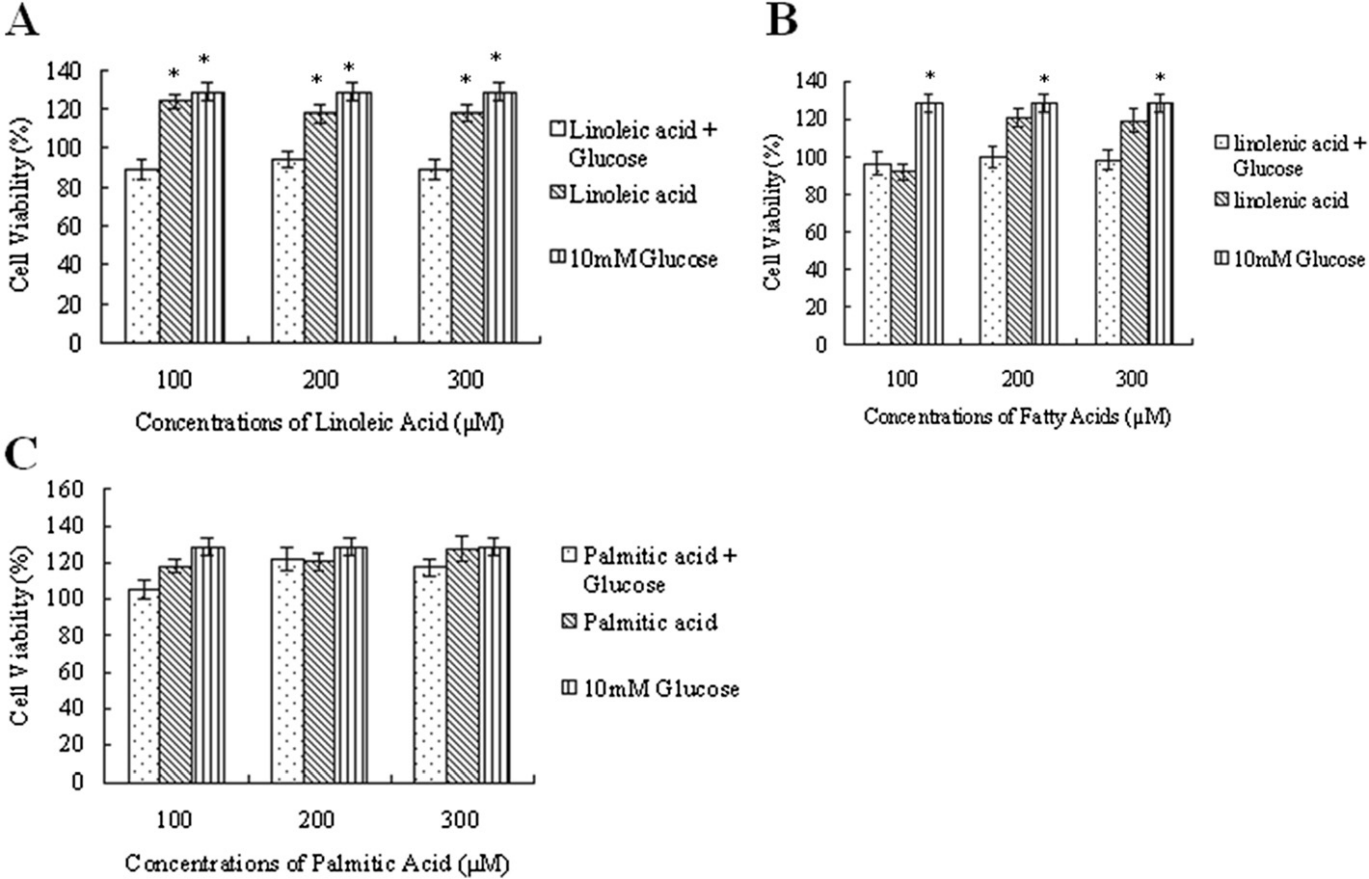
**Effect of different concentrations of fatty acids on the proliferation of RF/6A cells in the presence of 10 mM glucose.(A)**Linoleic acid **(B)** α-Linolenic acid **(C)** palmitic acid. *P < 0.05 compared to control.

Next the effect of EFAs: linoleic acid (LA) and α-linolenic acid (ALA) on the proliferation of RF/6A cells in the presence of varying concentrations of glucose was tested. It can be seen from Figure 2 that both LA and ALA significantly ameliorated glucose induced proliferation of cells to near normal, while saturated fatty acid: palmitic acid was ineffective. It is interesting to note that LA by itself enhanced while ALA suppressed the growth of RF/6A cells (Figure 2A and 2B). On the other hand, palmitic acid enhanced the growth of RF/6A cells though it was not statistically significant (Figure 2C). This suggests that possibly, only unsaturated fatty acids are capable of influencing enhanced proliferation induced by glucose. It is also likely that glucose and EFAs interact with each other to produce a metabolite of either glucose or EFAs or both (glucose and EFAs) that, in turn, suppresses the growth of RF/6A cells in vitro.

### Effect of EFAs on glucose-induced changes in mitochondrial succinate dehydrogenase enzyme activity

**Figure 3:**
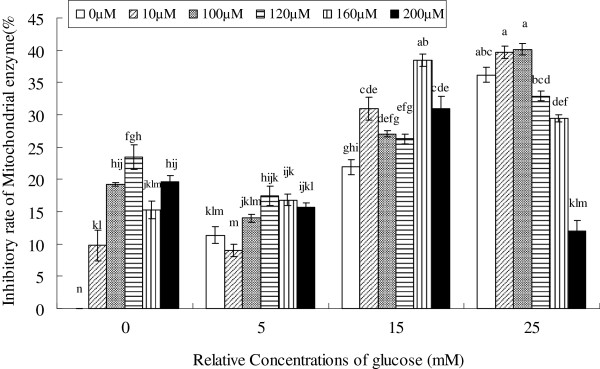
**Effect of various concentrations of ALA on glucose-induced changes in mitochondrial succinate dehydrogenase enzyme activity.** Data are mean ± S.D. Different superscript letters indicate significantly different means.

High glucose is known to be cytotoxic, partly by inducing mitochondrial stress [16,17]. Hence, we studied the effect of ALA on glucose-induced changes in mitochondrial succinate dehydrogenase enzyme activity as a marker of mitochondrial stress. A dose–response study showed that the activity of the enzyme was suppressed at all the concentrations of glucose (5, 15, 25 mM) significantly (Figure 3). With 25 mM glucose incubation for short time (48 hours), the activity decreased by 36.94%. Although ALA alone inhibited the activity of the enzyme by ~10- ~ 20%, it appeared to ameliorate glucose-induced suppression of succinate dehydrogenase activity by 25% (200 μM ALA co-treatment with 25 mM glucose).

### Effect of glucose, EFAs, and Zn on fluorescence polarization and microviscocity of RF/6A cells

**Figure 4:**
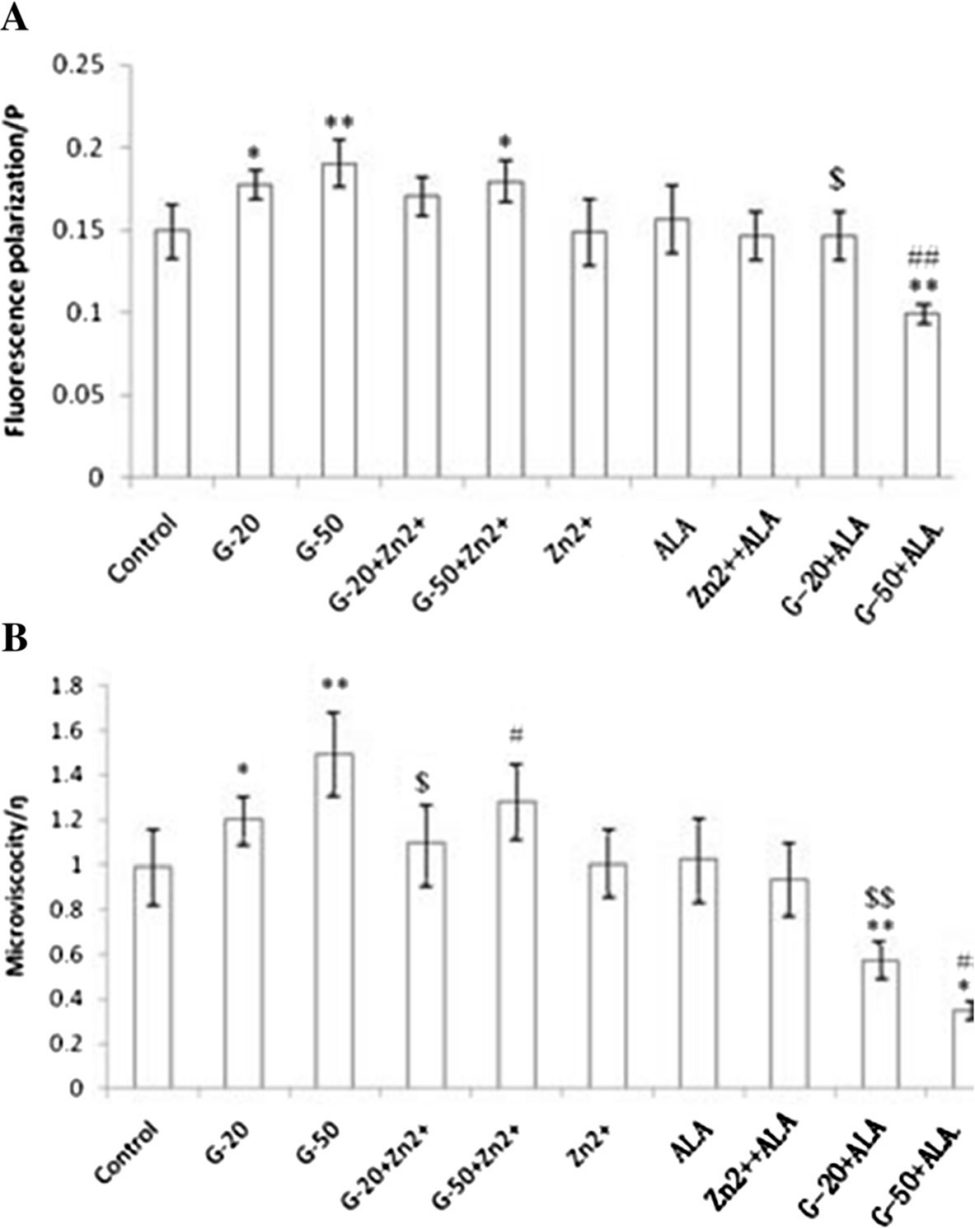
**Effects of the interactions of ALA with Zn**^**2+**^**on the fluorescence polarization and microviscocity of RF/6A cells.(A)** fluorescence polarization **(B)** microviscocity. Concentrations of Zn^2+^ and ALA were 80 and 3.7115 μmol/L respectively. Data are mean ± S.D. ^*^P < 0.05 and ^**^P < 0.01 compared to control. ^#^P < 0.05 and ^##^P < 0.01 compared to G-50. ^$^P < 0.05 and ^$$^P < 0.01 compared to G-20.

It is known that the “fluidity”-the thermal motion of the lipid bilayer of cell membrane-can be measured using fluorescence polarization technique- will give an indication as to cell membrane viscosity. The use of a fluorescent hydrocarbon, 1,6-diphenyl-1,3,5-hexatriene (DPH), to obtain fluorescence polarization and microviscosity values of the lipid bilayer of membranes of RF/6A cells treated with glucose, EFAs and Zn was performed. It is clear from these results given in Figure 4 that glucose enhanced fluorescence polarization and microviscocity (20 and 50 mM; 50 mM > 20 mM) that reverted to normal by treatment with Zn^2+^ and ALA. ALA was more potent than Zn^2+^ in reverting enhanced fluorescence polarization and microviscocity changes induced by glucose. On the other hand, Zn^2+^ and ALA by themselves or when added together did not have any effect on fluorescence polarization and microviscocity of RF/6A cells.

### Effect of glucose and EFAs on oxidative stress and anti-oxidant content of RF/6A cells

**Figure 5:**
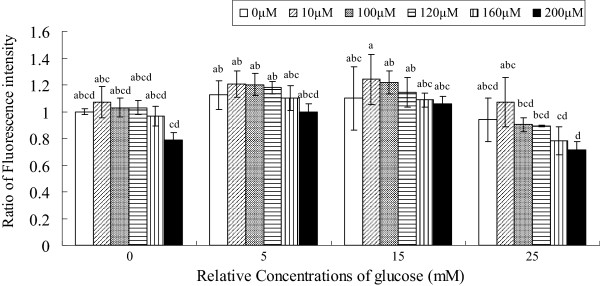
**Effect of glucose and ALA on the generation of reactive oxygen species in RF/6A cells.** Data are mean ± S.D. Comparisons were performed using Turkey’s post hoc test. Different superscript letters indicate significantly different means.

It is known that hyperglycemia produces oxidative stress by enhancing free radical generation in the cells [18,19]. Hence, we studied the effect of glucose on reactive oxygen species generation by RF/6A cells in vitro. The results shown in Figure 5 suggest that ROS generation was slightly increased by high glucose (with no significance), regardless of the concentrations tested. However, ALA showed a tendency to suppress ROS generation induced by glucose. The amount of ROS generation showed a downward trend as the concentration of ALA is increased (ALA concentrations tested: 10, 100, 120 and 200 μM). The maximum inhibitory was almost up to 30%. In general, ALA suppressed ROS generation to a significant degree in a dose dependent fashion.

**Figure 6:**
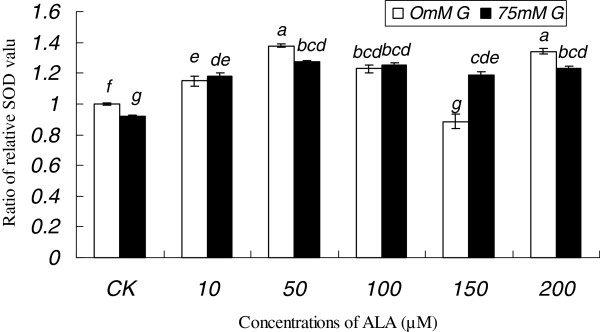
**Effect of glucose and ALA on the activity of SOD in RF/6A cells.** Data are mean ± S.D. Comparisons were performed using Turkey’s post hoc test. Different superscript letters indicate significantly different means.

Oxidative stress occurs in the cells, because of an imbalance between the prooxidant/antioxidant systems. Exposure of RF/6A cells to high amounts of glucose enhanced SOD activity compared to the control (Figure 6) suggesting that RF/6A cells are trying to compensate for the oxidative stress induced by excess glucose. ALA alone increased SOD activity to a significant degree in a dose dependent fashion. In the presence of excess of glucose, ALA maintained enhanced SOD activity. In fact, RF/6A cells exposed to the highest concentration of glucose showed higher activity of SOD in the presence of ALA compared to the control, suggesting that ALA may have the ability to balance enhanced oxidative stress by augmenting SOD levels in the cells.

### Effects of high glucose and ALA on the VEGF secretion by RF/6A cells

**Figure 7:**
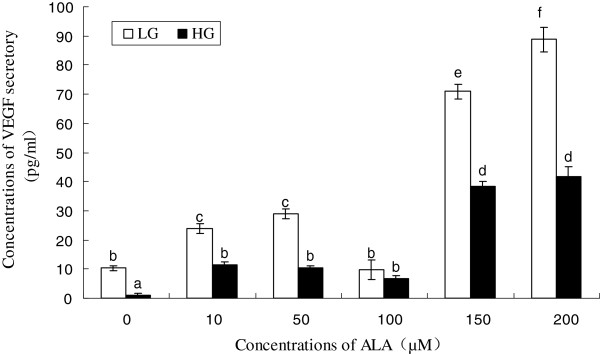
**The effect of high glucose and ALA on the VEGF secretory of RF6A cell line.** Data are mean ± S.D. Comparisons were performed using Turkey’s post hoc test. Different superscript letters indicate significantly different means.

It is evident from the results shown in Figure 7 that ALA by itself enhances the secretion of VEGF by RF/6A cells. Even glucose, at high concentrations augmented VEGF secretion by RF/6A cells as expected. In contrast to this, ALA decreased high-glucose induced VEGF secretion, suggesting that when there is a stimulus that normally enhances VEGF secretion, it will be kept in check by ALA.

## Discussion

In the present study, we observed that high glucose enhances proliferation of retinal endothelial cells. In experimental animals with diabetes, biochemical evidence of glucose toxicity can be found in the retinal and corneal layers that could be related to the morphological and physiological diabetic pathologies of the retinal and corneal cells. Enhanced intracellular glucose leads to augmentation of polyol pathway activity that results in an accumulation of intracellular sorbitol, which can be oxidized to fructose. Accompanying the alterations of glucose metabolism are disturbances in myoinositol and Na^+^ handling by the affected structures. As a result of these metabolic disturbances, corneal endothelium and the retinal vascular endothelial cells are destroyed leading to diabetic eye complications including retinopathy [20-22]. It is believed that hyperglycaemia causes diabetic complications by increasing polyol pathway flux; increased advanced glycation end-product (AGE) formation; activation of protein kinase C (PKC) isoforms; and increased hexosamine pathway flux [23]. It was noted that all above described mechanisms are activated by a single upstream event namely: mitochondrial overproduction of reactive oxygen species (ROS) [24]. Recently, it was opined that increased expression of the receptor for AGEs and its activating ligands play a significant role in hyperglycemia-induced tissue damage [25]. In view of this, indentifying molecules that protect tissues against the toxic effects of persistent hyperglycemia need to be identified. Success in such an endeavor could be of benefit in the management of diabetic complications.

Oxidative damage and mitochondrial dysfunction are considered to be significant factors underlying the initiation and progression of cellular changes during diseases, especially in diabetic retinopathy [26]. Dutot *et al*[27] observed that high glucose induced ROS overproduction in retinal pigment epithelial cells (RPEs). In contrast, Busik et al [28] reported that high glucose does not augment ROS generation. These discrepancies in the results reported could be attributed to differences in the cell types used by these investigators. In the present study, we noted that high glucose produced insignificant increase in ROS generation by RF/6A cells. Besides, high glucose caused significant inhibition of mitochondrial enzyme activity (Figure 3). Succinate dehydrogenase deficiency affects mitochondrial complex II, which links the TCA cycle with the electron transport chain by coupling the conversion of succinate to fumarate to the quinone pool [29]. The mechanism by which hyperglycemia causes an increase in mitochondrial ROS is not fully understood though it has been suggested that this could be due to enhanced production of pro-inflammatory cytokines in response to hyperglycemia. This is supported by the observation that exposure of HRECs to proinflammatory cytokines such as interleukin (IL)-1β (IL-1β) or tumor necrosis factor-α (TNF-α) led to increase in glucose consumption, augmented mitochondrial superoxide production, extracellular signal-related kinase (ERK) and Jun NH(2)-terminal kinase (JNK) phosphorylation, tyrosine phosphorylation, nuclear factor-kappaB (NF-kappa B) activation, and caspase activation. These results imply that HRECs respond to cytokines rather than to high glucose, and in an in vivo situation diabetes-related endothelial injury in the retina could be due to glucose-induced cytokine release by other retinal cells and not a direct effect of high glucose [28].

Oxidative stress plays an important role in the etiology of diabetic retinopathy. At the retina level, free radicals may preferentially react with the membrane polyunsaturated fatty acids leading to the release of lipoperoxide radicals. These lipoperoxides can induce damage to neuronal membranes that may affect vision [30]. The mechanism(s) by which n-3 PUFAs reduce oxidative damage and restore free radical homeostasis despite that fact that they are long-chain fatty acids with high degree of unsaturated and so are more likely to give rise to lipid peroxides is not completely understood though it is known that ALA, EPA, and DHA may reduce oxidative damage [31]. Besides, there is conflict with regard to the ability of n-3 PUFAs on the activity of antioxidant enzymes: some studies suggesting an increase while others reporting a decrease in the activities of antioxidant enzymes *in vivo*[32]. Few human studies have addressed the role of ALA in oxidative stress. According to the results of the present study, it is evident that ALA has, to some extent, the ability to restore the balance between prooxidant/antioxidant systems in cells that are exposed to oxidative stress. The results suggested that ALA suppressed ROS generation by enhancing SOD activity. It is possible that enhanced SOD activity could inhibit diabetes-induced increase in mitochondrial O_2_^·−^ and restore mitochondrial function to normal and thus, prevent vascular pathology.

EFAs form an important constituent of cell membranes and thus, play a critical role in maintaining their physical properties and consequently regulate cell functions [33]. Optimal membrane function requires a fluid state of the membrane, and this fluidity is largely dependent on its lipid composition that, in turn, depends on the unsaturated fatty acid(s) content and the ratio between unsaturated fatty acids and cholesterol/ phospholipids [34]. In the present study, we observed that glucose enhanced fluorescence polarization and microviscocity that reverted to normal by treatment with Zn^2+^ and EFAs. EFAs were more potent that Zn^2+^ in reverting to normal the enhanced fluorescence polarization and microviscocity changes induced by glucose. The fluidity is inversely related to fluorescence polarization; thus, the higher the polarization, the lower the fluidity, and vice versa. Hashimoto [34] found out that despite being members of the same n-3 family, DHA showed a higher increase in plasma membrane (PM) fluidity relative to that of EPA compared with the fluidity of control cell PM. The differences in the in situ three-dimensional structure in the bilayer leaflet (cross-sectional area per fatty chain and motional freedom along the long axis of the acyl chain between EPA and DHA) might be involved in their differential effect on membrane fluidity. Recently, while studying the effects of fatty acids with different unsaturations (from 0 to 6 double bonds) on membrane fluidity it was noted that fatty acids with 3 or less double bonds including SA, OA, LA and ALA had no effects on membrane fluidity, but only fatty acids with at least 4 or more double bonds including AA, EPA and DHA increased membrane fluidity [33]. As we noted that zinc enhanced the actions of EFAs on the fluidity and as it (zinc) is essential for the activity of Δ^6^ desaturase that converts ALA to its longer metabolites such as EPA and DHA, we propose that ALA is converted to form EPA and DHA that, in turn, is producing the changes in fluorescence polarization and microviscocity of RF/6A cells in the presence of glucose. But, this needs to be confirmed in future studies.

Pathological retinal neovascularization is the foremost destructive manifestation of diabetic retinopathy. VEGF is thought to play a significant role in pathological retinal neovascularization. Polyunsaturated fatty acids: AA, EPA and DHA, and their products lipoxins, resolvins, and protectins play an important role in the pathogenesis of pathological retinopathy in view of their anti-inflammatory; wound healing, and neuroprotective actions. Lipoxins, resolvins and protectins prevented hyperoxia-induced retinopathy in experimental animals suggesting that they are useful in the prevention and treatment of destructive angiogenesis [35,36]. Although progress has been made in understanding the protective properties of n-3 PUFAs [35-38], we still do not know exactly which specific lipid-processing pathways and which molecules govern these effects. Besides, it is not clear whether ALA as the precursor of EPA and DHA and their products such as lipoxins, resolvins and protectins has such retinoprotective function. According to the results of the present study, ALA enhanced the secretion of VEGF to a significant degree by RF/6A cells in vitro. In contrast, in the presence of high glucose levels, ALA inhibited VEGF secretion by RF/6A cells. These contrasting actions of ALA could be explained by suggesting that the protective action of ALA against retinal neovascularization is mediated, in part, through the formation of bioactive lipid mediators such as resolvins, lipoxins and protectins from the downstream product of ALA such as EPA and DHA in RF/6A cells [39]. It is known that the mechanism of the retino-protective action of DHA is due to the formation of its metabolite, 4-hydroxy-docosahexaenoic acid (4-HDHA), by the enzyme 5-lipoxygenase (5-LOX) that has potent antiangiogenic effect [40]. 4-HDHA acts via peroxisome proliferator-activated receptor γ PPARγ)to directly inhibit the sprouting and proliferation of endothelial cells. But 5-LOX is expressed by circulating leukocytes but not retinal cells. It is not yet certain whether ALA can be converted to DHA in an in vitro situation by RF/6A cells so that the latter (DHA) could be used to form 4-HDHA to produce the anti-angiogenic actions of ALA. This proposal needs to be verified in future studies. Furthermore, *in vivo* studies need to be performed to confirm the in vitro results obtained in the present study and confirm the above proposals.

## Conclusions

The results obtained in the present study suggest that glucose-induced changes in the growth of RF/6A cells, mitochondrial enzyme activity, fluorescence polarization and microviscocity of RF/6A cells and increased generation of free radicals in the cells can be suppressed by EFAs suggesting that they (EFAs) are of benefit in the prevention of high glucose toxicity to RF/6A cells.

## Methods

### Cell Culture

Rhesus macaque choroids-retinal endothelial cells (RF/6A) were used for this study, obtained from Institute of Biochemistry and Cell Biology, Chinese Academy of Sciences (Shanghai, China). RF/6A cells were cultured in DMEM (GIBCO) supplemented with 10% fetal bovine serum v/v and 100U/ml penicillin and 100 U/ml streptomycin in an atmosphere of 5% CO_2_ at 37°C. The cells were maintained with a medium change every 24–48 h, before being used in experiments. RF/6A cells (passage 4–12) were used in the following experiments.

### Effect of different concentration of glucose on RF/6A cells

Cells were seeded in each well of 96-well plate at a density of 1 × 10^4^ cell/well and incubated under different concentrations (5, 10, 20, 30, 40 and 50 mM) of glucose for 24, 48, 72 hours. Normal DMEM culture was used as a control. Thereafter, 20μL of 5 mg/mL MTT (3-[4,5-dimethythiazol-2-yl]-2,5- diphenyltetrazolium bromide) was added, and the cells were incubated for 4 h (to allow the formation of formazan precipitate, which subsequently was dissolved in dimethyl sulfoxide). The absorbance in each well was then measured with a microplate reader at 490 nm.

### Estimation of the activity of mitochondrial succinate dehydrogenase

Cells were plated at a density of 1 × 10^5^ cells/ml in 6-well plates for 24 h and then incubated with different concentrations of glucose (5,15,25 mM) and fatty acids (α- linolenic Acid) (10, 100, 120, 160, 200 μM) for 48 h to study their effect on these cells and thereafter, 200μL 5 mg/mL MTT was added to each well and cultured for an additional 4 h. Normal DMEM culture was used as a control. Then the cells were collected into PBS. The cell suspension was centrifuged for 10 min at 3000 rpm then supernatant were abandoned. The cells were suspended in 0.4 ml acidic isopropyl alcohol. After 20 min of standing, the absorbance of supernatant was measured with a microplate reader at 570 nm. The inhibitory rate of the mitochondrial enzyme (%) = [Absorbency_(Control)_-Absorbency_(Sample)_] × 100 %/Absorbency _(Control)_.

### Fluorescence polarization measurements

Cells were treated with different concentrations of glucose(20,50 mM), Zn^2+^ (80 μM), and FAs(3.7115 μM) for 24 h, and then digested by trypsin into a single cell suspension and harvested by centrifuging at 3000 rpm for 5 min at 4°C. Normal DMEM culture was used as a control. The cells were suspended in PBS and incubated with 1, 6-diphenyl1-1,3,5-hexatriene (DPH) in dark at 37°C for 30 min to allow complete incorporation of the probe into the membranes. Fluorescence measurements were performed on a Fluorescence Spectrophotometer (HITACHI F-4600). The excitation and emission wavelengths for DPH were selected with monochromators set to 359 nm (2.5 nm slit width) and 430 nm (2.5 nm slit width), respectively. The membrane fluidity was determined from fluorescence polarization (*P*) measurements. Fluorescence polarization (*P*) was calculated according to the following equation:

(1) $$\text{p}=\left(\text{Ivv}-\text{GIvh}\right)/\left(\text{Ivv}+\text{GIvh}\right)$$

(2) G=Ihv/Ihh

(3) $$\eta =2\text{P /}\left(0.46-\text{P}\right)$$

(4) $$\text{r}=\left(\text{Ivv}-\text{GIvh}\right)/\left(\text{Ivv}+2\text{GIvh}\right)$$

*IVV and Ivh* are the fluorescence intensities of the emitted light polarized parallel and vertical to the excitation light, respectively, and *G* is the grating correction factor.

### Determination of intracellular reactive oxygen species (ROS)

The measurement of intracellular ROS was based on the ROS-mediated conversion of non-fluorescent DCFH-DA into DCFH which is membrane impermeable. ROS oxidize DCFH to the brightly fluorescent compound 2,7-dichlorofluorescein (DCF). The intensity of fluorescence reflects the level of oxidative stress. Intracellular ROS was measured after incubation in the test medium by replaced the medium with non-serum DMEM medium (10 μmol/L DCFH-DA) and incubated in the dark for 40 min at 37°C. The level of DCFH fluorescence was measured at an excitation wave length of 488 nm and an emission wavelength of 525 nm by SpectraMax M5, Molecular Devices. The cells were treated with different concentrations of glucose (5, 15, 25 mM) and fatty acids (α- linolenic Acid) (10, 100, 120, 160, 200 μM) for 48 h and the amount of ROS production in the cells was detected with DCFH-DA method, using ROS detection kit (Beyontime Company, China). Normal DMEM culture was used as a control.

### Estimation of SOD activity

Cells were plated at a density of 1 × 10^5^ cells/ml in 6-well plates for 24 h. After treatment of glucose(75 mM) and ALA(10, 50, 100, 150, 200 μM), (Normal DMEM culture was used as a control) the activity of SOD were measured as described protocols of commercial reagent kits purchased from Nanjing Jiancheng Bioengineering Institute (Nanjing, China). The SOD activity were represented as corresponding value: Corresponding SOD concentration (%) = C_(sample)_/C_(control)_.

### Determination of VEGF content

RF/6A cells were seeded in each well of 6-well plate at a density of 3 × 10^5^ cell/well and incubated for 24 hours and then treated by the concentrations of 75 mM glucose and 10, 50, 100, 150, 200 μM ALA for 84 hours, respectively. Normal DMEM culture was used as a control. Cell culture fluid were collected and centrifuged for 5 min at 1500 rpm (4°C). The supernatant were assayed by VEGF ELISA kit according to the manufacturer’s instructions (Boster, China). The standard range for VEGF detection in this kit is 15.6-1000 pg/ml. The sensitivity is less than 1 pg/ml. Briefly, the samples of supernatants were double-diluted. 100 μl of each sample was added to each well coated with monoclonal detective antibody and were incubated for 90 min. After washing with PBS, biotin labeled antibody was added to bind to the cytokine. After 60 min incubation and washing, avidin-biotin-peroxidase complex ABC was added. 30 min later, a chromogenic substrate was added and the absorbance of each well was measured at 450 nm. The concentrations of VEGF were determined by interpolating from standard curves obtained with known concentrations of standard protein.

### Statistical analysis

The results are means ± S.D. Each experiment was performed at least in triplicate. Analysis was performed by one-way ANOVA with Tukey’s post hoc test, using SPSS 15.0 software for Windows. Differences among treatments with a value of P < 0.05 were considered to be statistically significant. Different superscript letters indicate significantly different means.

## Abbreviations

PUFAs, Polyunsaturated fatty acids; ALA, α-linolenic acid; DHA, Docosahexaenoic acid; EPA, Eicosapentaenoic acid; MTT, 3-(4, 5-dimethylthiazolyl-2)-2,5-diphenyltetrazolium bromide; ROS, Reactive oxygen species.

## Competing interests

The authors declare that there are no competing interests.

## Authors’ contributions

SS conceived the experimental design and performed the proofreading of manuscript. SS performed the experiments and the statistical analysis. SS wrote the manuscript. All authors discussed analyses and interpretation, read and approved the final manuscript.

This project was supported by SITP of Tongji University and Shanghai under-graduated student innovation project (No.1500-107-043).

## References

[B1] Cagliero E Maiello M Boeri D Roy S Lorenzi M Increased expression of basement membrane components in human endothelial cells cultured in high glucose J Clin Invest 1988 82 735 738 10.1172/JCI113655 3403725 PMC303571

[B2] Mandarino LJ Current hypotheses for the biochemical basis of diabetic retinopathy Diabetes Care 1992 15 1892 1901 10.2337/diacare.15.12.1892 1464244

[B3] Huang Q Sheibani N High glucose promotes retinal endothelial cell migration through activation of Src, PI3K/Akt1/eNOS, and ERKs Am J Physiol Cell Physiol 2008 295 C1647 1657 10.1152/ajpcell.00322.2008 18945941 PMC2603562

[B4] Lee TS Saltsman KA Ohashi H King GL Activation of protein kinase C by elevation of glucose concentration: proposal for a mechanism in the development of diabetic vascular complications Proc Natl Acad Sci USA 1989 86 5141 5145 10.1073/pnas.86.13.5141 2740348 PMC297573

[B5] Puskas LG Bereczki E Santha M Vigh L Csanadi G Spener F Ferdinandy P Onochy A Kitajka K Cholesterol and cholesterol plus DHA diet-induced gene expression and fatty acid changes in mouse eye and brain Biochimie 2004 86 817 24 10.1016/j.biochi.2004.10.004 15589691

[B6] Suresh Y Das UN Protective action of arachidonic acid against alloxan-induced cytotoxicity and diabetes mellitus Prostaglandins Leukotrienes Essential Fatty Acids 2001 64 37 52 10.1054/plef.2000.0236 11161584

[B7] Suresh Y Das UN Differential effect of saturated, monounsaturated, and polyunsaturated fatty acids on alloxan-induced diabetes mellitus Prostaglandins Leukot Essen Fatty Acids 2006 74 199 213 10.1016/j.plefa.2005.11.006 16412622

[B8] Suresh Y Das UN Long-chain polyunsaturated fatty acids and chemically-induced diabetes mellitus: Effect of ω-6 fatty acids Nutrition 2003 19 93 114 10.1016/S0899-9007(02)00856-0 12591540

[B9] Suresh Y Das UN Long-chain polyunsaturated fatty acids and chemically-induced diabetes mellitus: Effect of ω-3 fatty acids Nutrition 2003 19 213 228 10.1016/S0899-9007(02)00855-9 12620523

[B10] Das UN Suresh Y Huang Y-S, Ziboh VA Prevention of alloxan-induced cytotoxicity and diabetes mellitus by gamma-linolenic acid and other polyunsaturated fatty acids both in vitro and in vivo γ-Linolenic acid: Recent Advances in Biotechnology and Clinical Applications 2000 AOCS Press, Champaign, Illinois 112 125

[B11] Jayasooriya AP Ackland ML Mathai ML Sinclair AJ Weisinger HS Weisinger RS Halver JE Kitajka K Puskas LG Perinatal omega-3 polyunsaturated fatty acid supply modifies brain zinc homeostasis during adulthood Proc Natl Acad Sci USA 2005 102 7133 7138 10.1073/pnas.0502594102 15883362 PMC1129140

[B12] Bettger WJ Reeves PG Moscatelli EA Reynolds G O'Dell BL Interaction of zinc and essential fatty acids in the rat J Nutr 1979 109 480 488 430250 10.1093/jn/109.3.480

[B13] Manku MS Horrobin DF Karmazyn M Cunnane SC Prolactin and zinc effects on rat vascular reactivity: possible relationship to dihomo-gamma-linolenic acid and to prostaglandin synthesis Endocrinology 1979 104 774 779 10.1210/endo-104-3-774 436735

[B14] Ayala S Brenner RR Essential fatty acid status in zinc deficiency. Effect on lipid and fatty acid composition, desaturation activity and structure of microsomal membranes of rat liver and testes Acta Physiol Lat Am 1983 33 193 204 6673505

[B15] Cunnane SC Horrobin DF Manku MS Essential fatty acids in tissue phospholipids and triglycerides of the zinc-deficient rat Proc Soc Exp Biol Med 1984 177 441 446 6440147 10.3181/00379727-177-41970

[B16] Russell JW Golovoy D Vincent AM Mahendru P Olzmann JA Mentzer A Feldman EL High glucose-induced oxidative stress and mitochondrial dysfunction in neurons FASEB J 2002 16 1738 1748 10.1096/fj.01-1027com 12409316

[B17] Kumar S Kain V Sitasawad SL High glucose-induced Ca(2+) overload and oxidative stress contribute to apoptosis of cardiac cells through mitochondrial dependent and independent pathways Biochim Biophys Acta 2012 1820 907 920 10.1016/j.bbagen.2012.02.010 22402252

[B18] Kang BP Frencher S Reddy V Kessler A Malhotra A Meggs LG High glucose promotes mesangial cell apoptosis by oxidant-dependent mechanism Am J Physiol Renal Physiol 2003 284 F455 F466 12419773 10.1152/ajprenal.00137.2002

[B19] Yano M Hasegawa G Ishii M Yamasaki M Fukui M Nakamura N Yoshikawa T Short-term exposure of high glucose concentration induces generation of reactive oxygen species in endothelial cells: implication for the oxidative stress associated with postprandial hyperglycemia Redox Rep 2004 9 111 116 10.1179/135100004225004779 15231066

[B20] Marano CW Matschinsky FM Biochemical manifestations of diabetes mellitus in microscopic layers of the cornea and retina Diabetes Metab Rev 1989 5 1 15 10.1002/dmr.5610050102 2649333

[B21] Amano S Yamagishi S Kato N Inagaki Y Okamoto T Makino M Taniko K Hirooka H Jomori T Takeuchi M Sorbitol dehydrogenase overexpression potentiates glucose toxicity to cultured retinal pericytes Biochem Biophys Res Commun 2002 299 183 188 10.1016/S0006-291X(02)02584-6 12437967

[B22] Lorenzi M The polyol pathway as a mechanism for diabetic retinopathy: attractive, elusive, and resilient Exp Diabetes Res 2007 2007 61038 18224243 10.1155/2007/61038 PMC1950230

[B23] Brownlee M Biochemistry and molecular cell biology of diabetic complications Nature 2001 414 813 820 10.1038/414813a 11742414

[B24] Brownlee M The pathobiology of diabetic complications: a unifying mechanism Diabetes 2005 54 1615 1625 10.2337/diabetes.54.6.1615 15919781

[B25] Giacco F Brownlee M Oxidative Stress and Diabetic Complications Circ Res 2010 107 1058 1070 10.1161/CIRCRESAHA.110.223545 21030723 PMC2996922

[B26] Jarrett SG Lewin AS Boulton ME Stratton RD, Hauswirth WW, Gardner TW The Role of Mitochondrial Oxidative Stress in Retinal Dysfunction Studies on Retinal and Choroidal Disorder 2012 Humana Press, 203 239 http://www.springerlink.com/j55mp36247108885/about/

[B27] Dutot M Tourrette V Fagon R Rat P New approach to modulate retinal cellular toxic effects of high glucose using marine epa and dha Nutr Metab 2011 8 3 9 10.1186/1743-7075-8-3 PMC3132705 21679392

[B28] Busik JV Mohr S Grant MB Hyperglycemia-induced reactive oxygen species toxicity to endothelial cells is dependent on paracrine mediators Diabetes 2008 57 1952 1965 10.2337/db07-1520 18420487 PMC2453610

[B29] Smith AC Robinson A A metabolic model of the mitochondrion and its use in modelling diseases of the tricarboxylic acid cycle BMC Syst Biol 2011 5 102 10.1186/1752-0509-5-102 21714867 PMC3152903

[B30] Catala A A synopsis of the process of lipid peroxidation since the discovery of the essential fatty acids Biochem Biophys Res Com 2010 399 318 323 10.1016/j.bbrc.2010.07.087 20674543

[B31] Poudyal H Panchal SK Diwan V Brown L Omega-3 fatty acids and metabolic syndrome: Effects and emerging mechanisms of action Prog Lipid Res 2011 50 372 387 10.1016/j.plipres.2011.06.003 21762726

[B32] Serini S Fasano E Piccioni E Cittadini ARM Calviello G Dietary n-3 Polyunsaturated Fatty Acids and the Paradox of Their Health Benefits and Potential Harmful Effects Chem Res Toxicol 2011 24 2093 2105 10.1021/tx200314p 21902224

[B33] Yang XG Sheng WW Sun GY Lee JC Effects of fatty acid unsaturation numbers on membrane fluidity and a-secretase-dependent amyloid precursor protein processing Neurochem Int 2011 58 321 329 10.1016/j.neuint.2010.12.004 21184792 PMC3040984

[B34] Hashimoto M Hossain MS Yamasaki H Yazawa K Masumura S Effects of Eicosapentaenoic Acid and Docosahexaenoic Acid on Plasma Membrane Fluidity of Aortic Endothelial Cells Lipids 1999 34 1297 1304 10.1007/s11745-999-0481-6 10652989

[B35] Das UN Polyunsaturated fatty acids in pathological retinal angiogenesis Current Nutrition Food Sci 2009 5 94 111 10.2174/157340109788185562

[B36] Das UN Pathological retinal angiogenesis and polyunsaturated fatty acids Agri FOOD Industry hi-tech 2008 19 44 49

[B37] SanGiovanni JP Chew EY The role of omega-3 long-chain polyunsaturated fatty acids in health and disease of the retina Prog Retin Eye Res 2005 24 87 138 10.1016/j.preteyeres.2004.06.002 15555528

[B38] Bazan NG The metabolism of omega-3 polyunsaturated fatty acids in the eye: the possible role of docosahexaenoic acid and docosanoids in retinal physiology and ocular pathology Prog Clin Biol Res 1989 312 95 112 2529559

[B39] Connor KM SanGiovanni JP Lofqvist C Aderman CM Chen J Higuchi A Hong S Pravda EA Majchrzak S Carper D Increased dietary intake of omega-3-polyunsaturated fatty acids reduces pathological retinal angiogenesis Nat Med 2007 13 868 873 10.1038/nm1591 17589522 PMC4491412

[B40] Sapieha P Stahl A Chen J Seaward MR Willett KL Krah NM Dennison RJ Connor KM Aderman CM Liclican E Carughi A Perelman D Kanaoka Y SanGiovanni JP Gronert K Smith LEH 5-lipoxygenase metabolite 4-HDHA is a mediator of the antiangiogenic effect of w-3 polyunsaturated fatty acids Sci Transl Med 2011 3 12 10.1126/scitranslmed.3001571 PMC3711031 21307302

